# Constructing Auxin-Inducible Degron Mutants Using an All-in-One Vector

**DOI:** 10.3390/ph13050103

**Published:** 2020-05-23

**Authors:** Aisha Yesbolatova, Yuichiro Saito, Masato T. Kanemaki

**Affiliations:** 1 Department of Chromosome Science, National Institute of Genetics, Research Organization of Information and Systems (ROIS), Yata 1111, Mishima, Shizuoka 411-8540, Japan; aisha.yesbolatova@nig.ac.jp (A.Y.); yu_saito@nig.ac.jp (Y.S.); 2 Department of Genetics, The Graduate University for Advanced Studies (SOKENDAI), Yata 1111, Mishima, Shizuoka 411-8540, Japan

**Keywords:** auxin-inducible degron, conditional protein depletion, gene knockout, expression vector

## Abstract

Conditional degron-based methods are powerful for studying protein function because a degron-fused protein can be rapidly and efficiently depleted by adding a defined ligand. Auxin-inducible degron (AID) is a popular technology by which a degron-fused protein can be degraded by adding an auxin. However, compared with other technologies such as dTAG and HaloPROTAC, AID is complicated because of its two protein components: OsTIR1 and mAID (degron). To simplify the use of AID in mammalian cells, we constructed bicistronic all-in-one plasmids that express OsTIR1 and a mAID-fused protein using a P2A self-cleavage sequence. To generate a HeLa mutant line for the essential replication factor MCM10, we transfected a CRISPR-knockout plasmid together with a bicistronic plasmid containing mAID-fused MCM10 cDNA. After drug selection and colony isolation, we successfully isolated HeLa mutant lines, in which mAID–MCM10 was depleted by the addition of indole-3-acetic acid, a natural auxin. The bicistronic all-in-one plasmids described in this report are useful for controlling degradation of a transgene-derived protein fused with mAID. These plasmids can be used for the construction of conditional mutants by combining them with a CRISPR-based gene knockout.

## 1. Introduction

Targeted protein degradation via the ubiquitin–proteasome (UPS) pathway is a new direction for drug discovery and is a powerful approach to the study of protein function in living cells. Heterobifunctional chemical degraders, such as proteolysis-targeting chimeras (PROTACs) [1,2] and specific and nongenetic inhibitors of apoptosis-protein-dependent protein erasers (SNIPERs) [3,4], are drawing attention because of the high expectation that they will produce next-generation drugs. However, when employing these methodologies for the functional characterization of a protein of interest (POI), a specific and efficient chemical degrader is required for each POI. To achieve targeted depletion more systematically for functional characterization, it is more feasible to employ a method based on a polypeptide tag (also called a degron) that induces protein degradation in the presence of a defined ligand. Furthermore, degron-based genetic technologies are useful for the validation of new target proteins in chemical degrader development [5].

**Figure 1:**
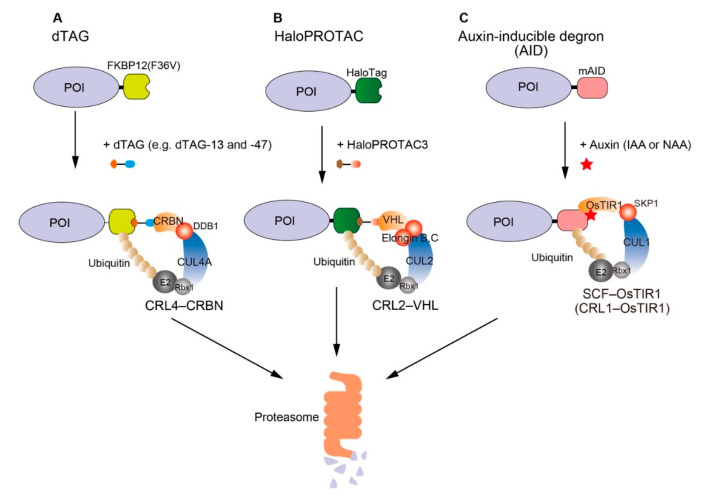
Schematic illustration of degron-based technologies for protein depletion in the presence of a defined ligand. (**A**) dTAG: a chemical degrader such as dTAG-13 and -47 binds a FKBP12(F36V)-fused POI and CRL4–CRBN, resulting in rapid degradation of the FKBP12(F36V)-fused POI via USP. (**B**) HaloPROTAC: a chemical degrader such as HaloPROTAC3 binds a HaloTag-fused POI, resulting in rapid degradation of the HaloPROTAC-fused POI via USP. (**C**) Auxin-inducible degron (AID): IAA or NAA binds OsTIR1, a component of SCF–OsTIR1. Subsequently, an mAID-fused POI is recognized for rapid degradation via UPS.

Researchers have explored the establishment of degron-based technologies by exploiting a defined chemical degrader that bridges a tag and an E3 ubiquitin ligase. An excellent example is dTAG, by which an FKBP12(F36V)-fused protein is recruited to CRL4–CRBN (CUL4A E3 ligase complexed with DDB1 and CRBN) in the presence of a chemical degrader such as dTAG-13 or -47 (Figure 1A) [6,7]. Another example is HaloPROTAC, by which a HaloTag-fused protein is recruited to CRL2–VHL (CUL2 E3 ligase complexed with elongin B/C and VHL) in the presence of a chemical degrader such as HaloPROTAC3 (Figure 1B) [8]. These degrader-based systems are composed of a single protein component, so that any protein fused with FKBP12(F36V) or HaloTag should be able to be induced for degradation by a defined degrader. For example, dTAG has been used to control a POI expressed from a transgene and to control an endogenous POI by directly fusing FKBP12(F36V) using CRISPR-based gene tagging [6,7,9,10].

We previously established another degron-based method, auxin-inducible degron (AID) technology (also known as auxin degron), by integrating a plant-specific degradation pathway into non-plant cells [11]. This is a two-protein component system, so two genetic modifications are required. A POI has to be fused with a 7-kD degron, called mini-AID (mAID) [12], and OsTIR1 (TIR1 derived from *Oryza sativa*) has to be expressed to form an E3 SKP1–CUL1–F-box ligase, SCF–OsTIR1 (also called CRL1–OsTIR1) (Figure 1C). In the presence of indole-3-acetic acid (IAA; a natural auxin) or 1-naphthaleneacetic acid (NAA; a synthetic auxin), the mAID-fused protein is recognized by SCF–OsTIR1 for rapid degradation via UPS. For this purpose, we previously established stable HCT116 and DLD1 cell lines expressing OsTIR1 [13,14]. Subsequently, we introduced an mAID-fused transgene or tagged an endogenous gene with mAID using CRISPR-based tagging. Although AID has been very popular for studying protein function because of rapid target depletion and its high efficiency [15,16,17], it was more laborious to employ AID than dTAG and HaloPROTAC. We wished to simplify the use of AID in mammalian cells, particularly in polyploid cells in which gene tagging of all alleles is more challenging than in diploid cells. For this purpose, we constructed a series of bicistronic plasmids encoding OsTIR1 and a POI fused with mAID. By introducing this plasmid, degradation of the mAID-fused protein can be induced using OsTIR1 expressed from the same plasmid. Furthermore, we show the generation of an AID mutant of polyploid HeLa cells by transfecting a CRISPR-knockout (CRISPR-KO) plasmid and the bicistronic plasmid for rescuing loss of the endogenous gene.

## 2. Results

**Figure 2:**
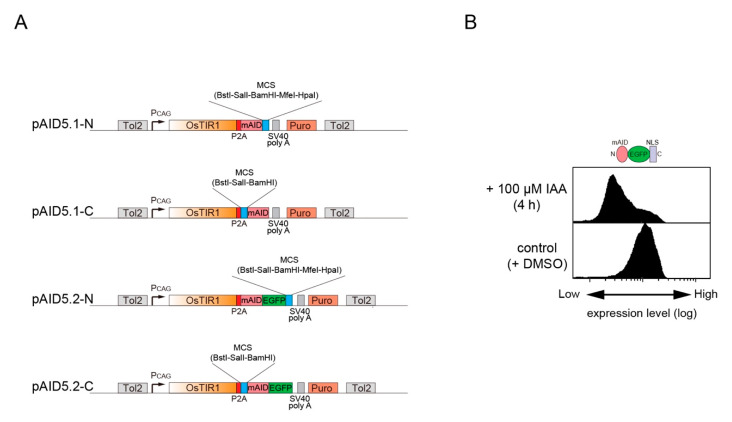
New all-in-one plasmids for controlling an mAID-fused protein. (**A**) Schematic illustration of pAID5.1-N/C and pAID5.2-N/C. A multi-cloning site (MCS) for cDNA cloning is shown in blue. (**B**) Reporter depletion in a clone derived from HCT116 cells. This clone was generated by introducing a plasmid based on pAID5.4-N. An mAID–EGFP–NLS reporter was detected after incubating the cells with or without 500 μM IAA for 4 h.

To control transgene expression, we constructed a series of bicistronic all-in-one plasmids (Figure 2A and Table 1). A CAG promoter drives expression of the first transgene, OsTIR1. The second expression unit is an mAID- or mAID–EGFP-fused transgene, which is connected to the upstream OsTIR1 gene via the P2A self-cleavage sequence [18]. Therefore, the two transgenes are transcribed as a single unit. Subsequently, OsTIR1 and an mAID-fused protein are cleaved during translation, resulting in expression of two independent proteins. These plasmids also contain TOL2 transposon sites and a puromycin- or hygromycin-resistant marker for stable integration to the genome and for selection of clones, respectively. To test the functionality of these plasmids, we cloned a nuclear localization signal (NLS) to pAID5.2-N to express OsTIR1 and mAID–EGFP–NLS. We transfected this plasmid together with pCS-TP, which expresses TOL2 transposase [19], to human HCT116 cells and subsequently established a stable clone. This clone was treated with or without 100 μM IAA for 4 h followed by flow cytometry to detect mAID–EGFP–NLS (Figure 2B). The reporter expression was clearly decreased in cells treated with 100 μM IAA, showing that the bicistronic plasmids are functional.

**Figure 3:**
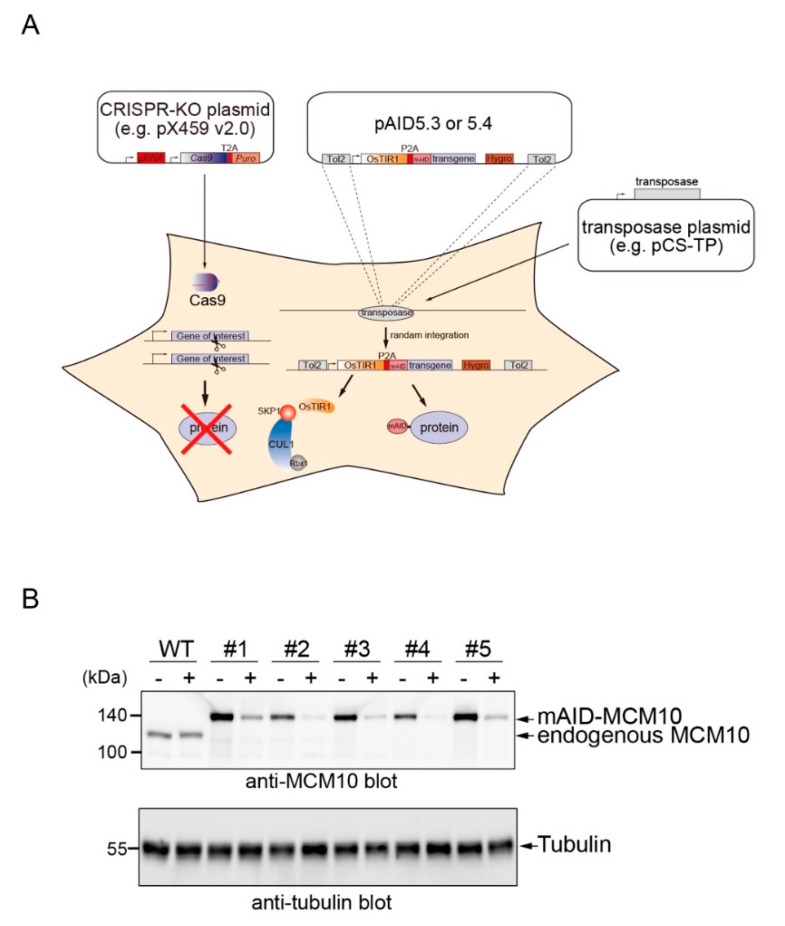
Generation of MCM10 mutant cell lines on a HeLa background. (**A**) Schematic showing the strategy used to construct an AID mutant using CRISPR-KO and bicistronic pAID5 plasmids. The CRISPR-KO plasmid expresses a Cas9–gRNA complex for gene knockout. The bicistronic rescue plasmid is integrated into the genome by the action of TOL2 transposase and expresses both OsTIR1 and mAID-fused protein. (**B**) Immunoblot showing endogenous MCM10 and mAID–MCM10. HeLa clones were treated with 500 μM IAA for 2 h and protein extracts were separated by SDS-PAGE. Tubulin is a loading control.

Next, we wished to generate a conditional HeLa mutant for a gene essential for cell viability. For this purpose, we chose a replication factor, MCM10, which is essential to initiate DNA replication in all eukaryotes [20,21,22,23]. The strategy is shown in Figure 3A. To knock out the endogenous gene expressing MCM10, we designed a CRISPR-KO plasmid that cleaves the 3′ splicing junction at exon 5 (ENSE00000999778). Importantly, this plasmid encodes a guide RNA (gRNA), Cas9, and a puromycin resistance gene [24]. Therefore, it is possible to efficiently kill un-transfected cells by transiently treating cells with puromycin after transfection. To rescue the loss of MCM10, we constructed a bicistronic rescue plasmid by cloning human MCM10 cDNA in pAID5.3-N. It should be noted that the MCM10 cDNA in this plasmid will not be recognized and cleaved by the Cas9–gRNA complex expressed from the CRISPR-KO plasmid. We transfected three plasmids, the CRISPR-KO plasmid, the pAID5.3-N-based rescue plasmid, and transposase encoding pCS-TP, into HeLa cells. After the transfection, the cells were transiently treated with puromycin and colonies were formed in the presence of hygromycin (see details in Materials and Methods). Isolated clones were grown and treated with or without 500 μM IAA for 2 h (Figure 3B). In all five clones, the transgene-derived mAID–MCM10 protein was expressed and the endogenous MCM10 protein was lost. It should be noted that MCM10 was essential for DNA replication, so that the mAID–MCM10 protein was functional for supporting DNA replication in these clones. Importantly, mAID–MCM10 was depleted by the IAA treatment. Thus, these MCM10 mutant lines generated by one transfection may be used for functional studies of MCM10.

## 3. Discussion

Here, we showed a series of all-in-one pAID5 plasmids that enable conditional degradation control of an mAID-fused POI (Figure 2). By using one of them, we demonstrated a method to generate an AID mutant of HeLa cells, in which MCM10 was conditionally degraded by adding IAA. It should be noted that the level of expression of mAID-MCM10 in clones 2 and 4 was more or less comparable to that in WT cells (Figure 3B). Because MCM10 is essential for viability, and multiple copies of the rescue plasmid can be integrated into the genome, clones having a sufficient level of mAID–MCM10 might have been positively selected during colony formation. In other words, it might be possible to select clones optimally expressing an mAID-fused POI by the strategy shown in Figure 3A. This strategy is less laborious than tagging an endogenous gene in all alleles, in particular when polyploid cell lines such as HeLa are used. It should be noted that the transcriptional regulation of an mAID-fused transgene is different from that of the endogenous gene. Therefore, the transgene cannot be controlled transcriptionally by endogenous biological processes, such as the cell-cycle control system. Additionally, there is a possibility that the mAID tag (7.4 kDa) might affect the function of a target protein.

We and others have previously constructed other bicistronic expression plasmids utilizing an internal ribosome entry site (IRES) and used them to control the degradation of mAID-fused POIs by AID [11,25]. A drawback of using an IRES is that expression of the second transgene located downstream of the IRES is very inefficient [26,27]. Therefore, the level of expression of OsTIR1 is much higher than that of an mAID-fused POI. This can be a potential problem because a high level of OsTIR1 causes leaky degradation even without IAA [13]. With the new P2A-based bicistronic plasmids, the levels of expression of OsTIR1 and an mAID-fused POI should be proportional because the cleavage efficiency of P2A is extremely high [18]. This feature should be advantageous for expression and degradation control of an mAID-fused POI.

## 4. Materials and Methods

### 4.1. Plasmids

The pAID5 plasmids are available from the addgene repository. The sequence information of these plasmids is available from the distributor. To construct a CRISPR-KO plasmid for targeting MCM10, two oligonucleotides (5′-CACCGACCGCAAGTACTACACCTGG-3′ and 5′-AAACCCAGGTGTAGTACTTGCGGTC-3′) were hybridized. The hybridized DNA was cloned at the *Bbs*I site of pX459 v2.0 (addgene, #62988) [24]. An expression plasmid encoding TOL2 transposase, pCS-TP, was previously described [19].

### 4.2. Cell Culture, Transfection, and Isolation of Clones

HCT116 cells were cultured in McCoy’s 5A medium, supplemented with 10% FBS, 2 mM glutamine, 100 U/mL penicillin, and 100 μg/mL streptomycin at 37 °C under an atmosphere of 5% CO_2_ in air. Transfection and isolation of HCT116 clones were conducted as previously described [14]. HeLa cells were cultured in DMEM, supplemented with 10% FBS, 100 U/mL penicillin, and 100 μg/mL streptomycin at 37 °C under an atmosphere of 5% CO_2_ in air. HeLa cells were seeded at 0.5 × 10^5^ cells per well in a 6-well plate. A plasmid mixture was prepared by mixing 8 μg of CRISPR–Cas9 KO plasmid, 200 ng of pMK5.3-N-based plasmid, and 200 ng of CS-TP. Opti-MEM (Thermo Fisher Scientific, #31985062) was added to bring the final volume to 50 μL. Subsequently, 4 μL of FuGENE HD Transfection Reagent (Promega, #E2311) was added. After incubating the transfection mixture for 15 min, the mixture was applied to the cells. One day after transfection, the cells were collected, resuspended in 2 mL of medium, and seeded in a 10-cm dish, which contained 10 mL of medium with 1 μg/mL puromycin. Two days later, the medium was exchanged with fresh medium containing 200 μg/mL hygromycin B without puromycin. The culture medium containing hygromycin B was exchanged every three to four days until colonies became visible. Colony formation took 19 to 22 days.

### 4.3. Flow cytometry

HCT116 cells were seeded at 1 × 10^5^ cells/well in a 6-well plate and grown for two days. To detect EGFP, cells were trypsinized and fixed in 4% methanol-free paraformaldehyde phosphate buffer at 4 °C overnight. Fixed cells were washed and resuspended in PBS containing 1% BSA. Flow cytometric analysis was performed on an Accuri C6 machine (BD Biosciences) using FCS Express 4 software (DeNovo Software, version 4). We analysed 10,000 cells from each sample.

### 4.4. Protein Detection by Western Blotting

HeLa clones were seeded at 1 × 10^5^ cells/well in a six-well plate and grown for one day. After treating with 500 μM IAA or DMSO vehicle control for 4 days, the treated cells were lysed in RIPA buffer (25 mM Tris-HCl, pH 7.6; 150 mM NaCl, 1% NP40, 1% sodium deoxycholate, and 0.1% SDS). After centrifugation to pellet the cells, the supernatant was mixed with 2 × SDS sample buffer (Tris-HCl, pH 6.8; 4% SDS, 20% glycerol, 10% 2-mercaptoethanol, and 0.004% bromophenol blue) before incubating the sample mixture at 95 °C for 5 min. Equal amounts of protein were loaded onto a 7.5% SDS-PAGE gel and transferred onto a Hybond ECL membrane (GE Healthcare). The membrane was incubated with anti-MCM10 antibodies (Proteintech, #1225-1-AP) at 4 °C overnight. Subsequently, it was incubated with anti-rabbit IgG antibodies conjugated with StarBright Blue 700 (Bio-Rad, #12004161; Hercules, CA, USA) and anti-tubulin antibodies conjugated with rhodamine (Bio-Rad, #12004165) at room temperature for 1 h. Fluorescent images were acquired with a ChemiDoc Touch MP system (Bio-Rad).

## 5. Conclusions

In summary, the bicistronic pAID5 plasmids described in this report should be useful for controlling the degradation of a transgene-derived POI fused with mAID. These plasmids can be used for the construction of conditional mutants by combining them with a CRISPR-based gene knockout.

## Figures and Tables

**Table 1:** It shows the features of pAID5 plasmids.

Plasmid	Tag	Marker	Transposon
pAID5.1-N	mAID	Puromycin	TOL2
pAID5.1-C	mAID	Puromycin	TOL2
pAID5.2-N	mAID–EGFP	Puromycin	TOL2
pAID5.2-C	mAID–EGFP	Puromycin	TOL2
pAID5.3-N	mAID	Hygromycin	TOL2
pAID5.3-C	mAID	Hygromycin	TOL2
pAID5.4-N	mAID–EGFP	Hygromycin	TOL2
pAID5.4-C	mAID–EGFP	Hygromycin	TOL2
